# Of ponds and people: Governance to balance biodiversity conservation and carp pond farming in Central Europe

**DOI:** 10.1007/s13280-025-02192-y

**Published:** 2025-05-06

**Authors:** Kathleen Schwerdtner Máñez, Irene Ring, Rosa Hildebrandt, Uwe Brämick

**Affiliations:** 1 International Institute Zittau, TUD Dresden University of Technology, Markt 23, 02763 Zittau, Germany; 2 Present Address: Baltic Sea Conservation Foundation, c/o Basislager, Richard-Wagner-Straße 1a, 18055 Rostock, Germany; 3 Institut für Binnenfischerei Potsdam-Sacrow, Im Königswald 2, 14469 Potsdam, Germany

**Keywords:** Biodiversity governance, Carp, Cultural landscapes, Environmental policy instruments, Pond aquaculture

## Abstract

Due to the loss of many natural water bodies, artificially created ponds often serve as refuge for numerous endangered species. The history of pondscapes in Central Europe is closely tied to the introduction of the common carp. Changing political, social, and climatic conditions, along with the increasing threat from fish-eating species, make the economic viability of pond aquaculture increasingly fragile. However, maintaining these pondscapes is crucial to meet societal demands for landscape and nature conservation. This article addresses the neglect of pondscapes in conservation literature and contributes to the ongoing discussion on the importance of cultural landscapes for biodiversity conservation. Lusatia, one of Europe’s largest pondscapes, faces challenges that reflect those encountered in other European pondscapes. In this study, we present these challenges along with the governance approaches implemented in Lusatia, using this analysis to outline potential solutions for conserving European pondscapes more broadly.

## Introduction

Traditionally, much of biodiversity conservation has been about limiting human impacts. Ideas of pristine nature and wilderness as valuable land free from human presence and untransformed by human action have long dominated conservation actions (Cronon 1995; Adams and Hutton 2007). But in an increasingly human-dominated world, this approach has reached its limits, and alternative solutions to address the current biodiversity crisis must be found. Cultural landscapes provide the opportunity to coproduce biodiversity through biodiversity-friendly and sustainable land-use schemes and are therefore seen as a promising conservation strategy (Kueffer and Kaiser-Bunbury 2014). Often characterised by semi-natural ecosystems, cultural landscapes can hold remarkable biological diversity (Barthel et al. 2013).

Literature on biodiversity conservation in cultural landscapes strongly focuses on terrestrial systems (Farina 2000; Assandri et al. 2018; García Ruiz et al. 2020). Relating to aquatic ecosystems, substantial knowledge has been accumulated on the biodiversity of natural lakes and other smaller natural water bodies. However, man-made ponds have been mostly overlooked (e.g. Brönmark and Hansson 2002), and there is a particular dearth of data regarding the biodiversity of semi-natural fishponds (Broyer and Curtet 2012). Despite the evidence presented in several papers that fishponds are important habitats for amphibians (Kloskowski 2010), macrophytes and breeding birds (Broyer and Curtet 2012), their general role in biodiversity conservation has long been neglected and undervalued. Like other artificially created water bodies, fishponds constitute unique ‘islands’ of aquatic biodiversity, providing stepping-stones and increased connectivity between freshwater habitats for a variety of taxa that are fully or partially aquatic. This phenomenon is especially evident in regions where floodplains and wetlands have disappeared due to changes in agriculture and infrastructure (EPCN 2008; Sayer et al. 2012; Bätzing 2013). Ponds have been found to support a higher species richness at the pond level and a greater biodiversity at the landscape level in comparison with other freshwater habitats (EPCN 2008; Wezel et al. 2014; Hill et al. 2017). They also deliver a multitude of ecosystem services, including provisioning services, regulating services (e.g. water retention, microclimate regulation and nutrient fixation) and cultural services (e.g. cultural identity, recreation and tourism) (Plieninger et al. 2013; Färber et al. 2020; Rey-Valette et al. 2024). As they help retain water during hot and dry spells and improve water quality flowing into other freshwater habitats, ponds offer effective nature-based solutions for climate change mitigation and adaptation (Biggs et al. 2024).

In Central Europe, pond construction for fish production dates back nearly 1000 years and is closely linked to the introduction of the common carp (*Cyprinus carpio*). Carp pond farming remained largely unaffected by intensification, except for a brief period in the late twentieth century, mainly in socialist countries, when high-density production methods were introduced. While intensive methods may come along with nutrient emission, sediment turbation and downstream transport, application of disinfectants, etc., traditional extensive carp pond systems are considered non-polluting and stabilising for ecosystems (Kestemont 1995; Kocour et al. 2025). Regular maintenance, including reed cutting and sediment removal, is essential for preventing overgrowth and enhancing biodiversity, especially for macrophytes and invertebrates (Sayer et al. 2012). Rare plants like moss grass (*Coleanthus subtilis*) benefit from regular pond draining (Richert et al. 2016).

Across Europe, changing political, social, and climatic conditions—such as environmental regulations, rising production costs, and water scarcity—threaten the economic viability of many carp pond farms (Wezel et al. 2014; Zdeněk et al. 2025). Additionally, the growing populations of protected species such as cormorants, herons, otters, and beavers increase production costs while also endangering and reducing fish production yields (Brämick and Schiewe 2023). With fewer enterprises and reduced pond areas, the continued existence of pondscapes is at risk. Current support measures are inadequate, and without proper maintenance, the existence and ecological functions of ponds are compromised. However, societal demands for landscape conservation and nature preservation, especially in meeting legal obligations to maintain specific habitat or population statuses, can only be fulfilled if pondscapes are properly maintained.

This article highlights European pondscapes as endangered cultural landscapes of significant conservation value. Focusing on the Lusatian pondscape as one of the largest artificially created pondscapes in Central Europe, we examine the challenges of maintaining carp farming, describe current governance approaches, and propose opportunities for improvement. By addressing the lack of attention to pondscapes in conservation literature, we expand the discussion on cultural landscapes and biodiversity conservation to include man-made water bodies. The article provides an overview of carp pond farming history and current challenges, followed by an assessment of governance approaches and potential improvements. While focused on Lusatia, our findings are relevant to other European pondscapes with similar histories and challenges, subject to European Union (EU) legislation.

## Carp pond farming in Central Europe

Carp ponds are shallow, nutrient-rich water bodies that are cyclically filled and drained. Fish production relies on the pond’s food web, supported by autotrophic and heterotrophic organisms. In Central Europe, under typical climate conditions, 250–350 kg/ha of carp can be produced per growing season, depending on nutrient availability from water inflows, sediment, and precipitation. The natural food supply can be enhanced with external nutrients (manure, fertiliser) and supplemented with feeds like grain or dry formulations, increasing seasonal carp production to 500–1000 kg/ha (Müller-Belecke 2023b). Market-sized carp requires three growing seasons, cultivated by year-class in ponds with varying size, morphometry, and management.

Carp aquaculture in China dates back 8000 years (Nakajima et al. 2019), but the belief that carp was introduced to Europe from China was disproven by Balon (1995), who showed that wild carp existed in the Danube catchment area. After the last ice age, carp spread from the Ponto-Caspian Basin, likely by human intervention. While Romans consumed carp from the Danube, there is no evidence they reared them (Hoffmann and Winiwarter 2010). Carp aquaculture developed in monastery ponds with the spread of Christianity, and as monasteries expanded, they introduced carp farming to new regions (Balon 1995; Beveridge and Little 2002). The spread of carp aquaculture was driven by broader economic and environmental factors (Hoffmann 1995).

By the tenth century, population growth led to increased cereal production, converting woodlands into plowland and altering hydrological conditions, along with a rise in water-powered grain mills. This caused nutrient emission and stressed freshwater fisheries as demand for food fish grew. Evidence of high fishing pressure on stocks in natural waters includes a decline in the average size of sturgeon (*Acipenser sturio*), salmonids, and pike perch (*Sander lucioperca*) in fish remains from Central European sites (Paul 1980; Hoffmann 1995). In response, the culture of warm-water species, particularly common carp, spread (Hartstock 2004; Hoffmann 1995, 2005). Pond construction also helped address fertiliser shortages by acting as nutrient traps, making fish pond farming a solution for infertile soils (Hoffmann and Winiwarter 2010).

In Central Europe, fish pond construction began in the eleventh century, expanding rapidly during the twelfth and thirteenth centuries, often in areas with poor or wet soils. By the thirteenth century, pond farming spread to Eastern Europe. By the early fourteenth century, standardised carp management practices were well-documented (Hoffmann 1995).

**Fig. 1:**
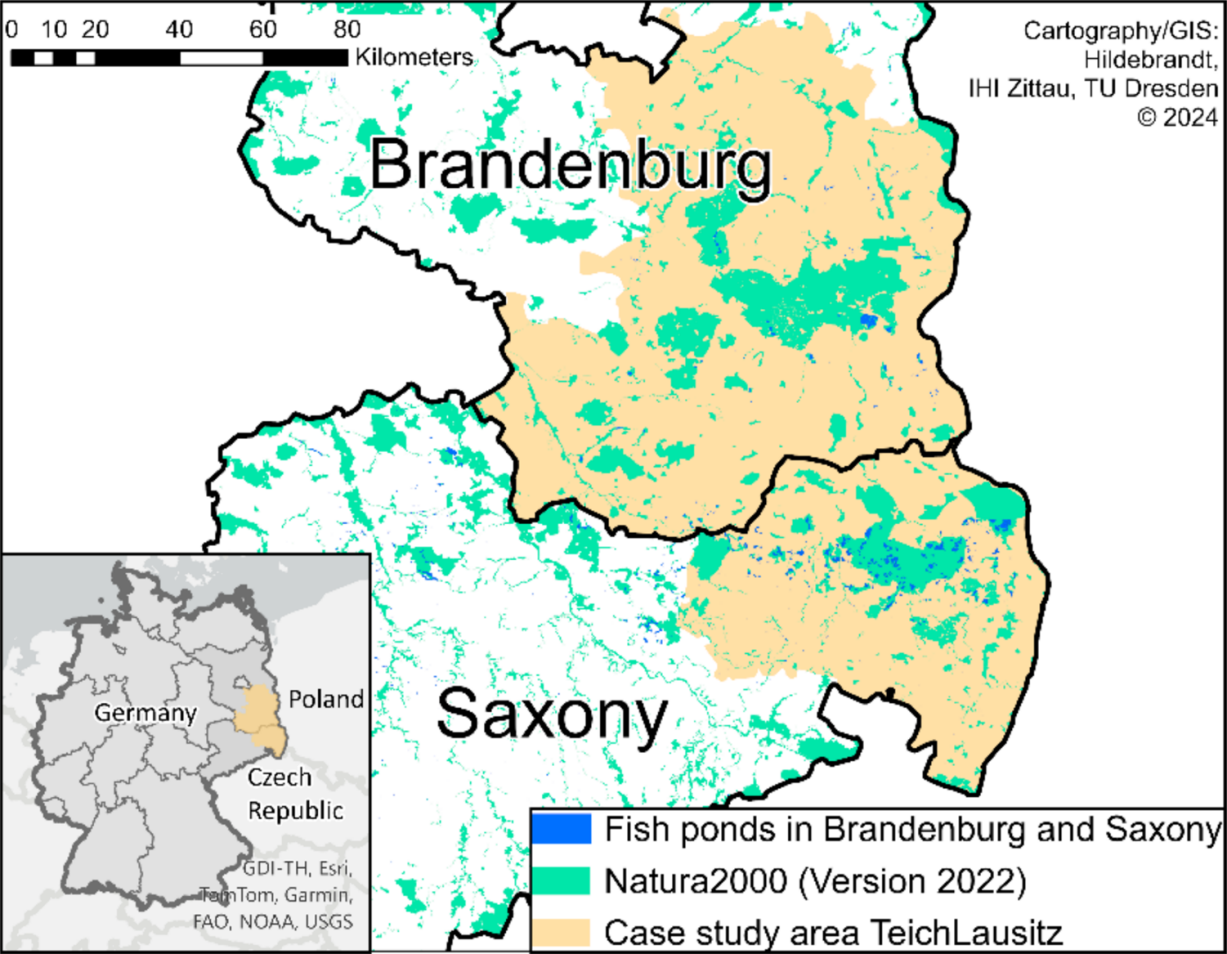
Lusatian pond landscape and Natura 2000 sites in Saxony and Brandenburg

Carp pond farming in Europe peaked between the fourteenth and sixteenth centuries, with carp prices six times higher than pigs (Bätzing 2013). Its decline began during the Little Ice Age due to unfavourable climate and accelerated with the introduction of mineral fertilisers in the 1850s. Fertilisation made pond-based nutrient collection obsolete, allowing agricultural expansion on previously pond-used soils (Szumiec and Augustyn 2000). A revival of carp aquaculture followed industrialisation and population growth, but declining economic feasibility has led to its continued decline. Despite significant losses, countries like Poland (70 000 ha) and the Czech Republic (41 000 ha) still have high pond densities (EUMOFA 2021; Turkowski 2021). One of the largest continuous pondscapes in Europe is in Lusatia, Germany, with approximately 1250 ponds covering about 9500 ha across Brandenburg and Saxony (Fig. 1).

## The Lusatian pondscape: Biodiversity hotspot and management challenges

### Environmental regulations and protected species

The Lusatian pondscape, spanning Saxony and Brandenburg, is a globally unique cultural landscape, including the UNESCO biosphere reserve Upper Lusatian Heath and Pond Landscape on the Saxon side. It is a biodiversity hotspot (Ackermann and Sachteleben 2012), with its ponds and ditches serving as key habitats for many bird and amphibian species (Ohnesorge et al. 2013). The area hosts priority species from the Habitats Directive, including the Eurasian otter (*Lutra lutra*) and European fire-bellied toad (*Bombina bombina*), and supports diverse insect populations, including 53 of Germany’s 80 dragonfly species, along with more than 20 native fish species (Böhnert et al. 1996).

Fishponds attract piscivorous species such as the Eurasian otter and the Great Cormorant (*Phalacrocorax carbo sinensis*). Once considered pests, these species have recovered due to European nature conservation directives like the Birds Directive (RL 79/409/EWG 1979), which protects all European wild birds and requires the designation of Special Protection Areas (SPAs). The cormorant is now more widespread than in the past 150 years, leading to significant environmental conflicts (Cowx 2013; Carss 2022). Other species, such as grey herons (*Ardea cinerea*) and great egrets (*Ardea alba*), have also increased, causing further damage to fish culture in ponds.

The majority of fishponds in Brandenburg and Saxony are protected under the 1992 Habitats Directive (RL 92/43/EWG) as Special Areas of Conservation (SACs), making them part of the Natura 2000 network. Within these sites, activities must not lead to habitat deterioration or disturb species, and managed ponds that harbour priority (Annex II) or rare species (Annex IV) require additional protection measures. Yet, while regular maintenance—such as reed cutting—is crucial for maintaining pond biodiversity, the European Commission provides no specific guidelines, and German conservation law restricts reed cutting during the growing season. This conflict between legal restrictions and necessary management practices challenges pond farmers to balance compliance with conservation needs and their economic interests (Ritterbusch et al. 2004).

### More complex, more costly: Changing production conditions

The reunification of Germany in 1990 was followed by an initial decline in demand for carp in Lusatia, as a greater variety of other species, such as salmon, trout, cod, and pollock, became more readily available than they had been under the socialist economy. Although this trend has come to a halt in recent years on a comparably low level, it was accompanied by a consistent decline in carp production figures. Yields now average around 200 kg/ha in Brandenburg and 220 kg/ha in Saxony. The reasons for this are a more extensive production with lower stocking densities because of nature conservation requirements and higher losses, particularly due to predators (Brämick and Schiewe 2024).

Research indicates that losses caused by disease, environmental factors, and predation have risen significantly due to growing populations of fish-eating animals. Average losses in the first growth season have increased from 70 to 80%, in the second season from 40 to 60%, and in the third from 10 to 30%. Maximum values now reach 94%, 92%, and 92%, respectively (Müller-Belecke 2023b). Increasing predation pressure in combination with recurrent shortages in young fish for stocking, rising prices for supplementary feedstuffs, fuel and electricity have significantly increased production costs. Since the liberalisation of Germany’s electricity market, electricity prices have surged by 210% between 1998 and 2024, while fuel prices have risen by 220% (Statistisches Bundesamt 2024).

Climate change further complicates carp production by increasing risks such as oxygen depletion due to higher temperatures in late summer. In winter, increased metabolism leads to higher energy demands, requiring more frequent feeding. Prolonged dry periods during summer can also cause ponds without a stable water supply to dry out (AG NASTAQ 2020).

In the last decade, the number of carp pond businesses in Germany decreased by 35%, impacting the Lusatian region as well (Brämick and Schiewe 2023). As of 2023, Brandenburg still had 22 pond farms, covering around 2949 ha, with farm sizes ranging from 50 ha to several hundred hectares (Brämick and Schiewe 2024). Most ponds are privately owned, with some leased to other pond farmers (Müller-Belecke 2023a), while a few have been transferred to the Brandenburg Nature Protection Fund Foundation and are pilot-tested to be managed according to nature conservation objectives without fish stocking (Stiftung Naturschutzfonds Brandenburg 2025). In Saxony, there were 119 pond farms covering 7713 ha (Brämick and Schiewe 2024). Most of the ponds in Saxony are state-owned and leased to farmers (LfULG 2023), with a few privately owned, and a small percentage owned by nature conservation associations.

Rising production costs and increased demand for regionally produced fish have led to higher average carp prices, from 5.05 €/kg in 2020 to 6.84 €/kg in 2023 (Statistisches Bundesamt 2024). In direct marketing, carp is sold at 6.84 €/kg, while wholesale prices range from 3.31 to 3.78 €/kg. Around 10% of fish in Saxony is marketed directly, compared to up to 40% in Brandenburg (Müller-Belecke 2023a). Growing demand may boost production, but nature conservation regulations restrict this potential. Economic studies in Lusatia (Huber 2021a, b), Bavaria (Lasner et al. 2020), and the Czech Republic (Zdeněk et al. 2025) consistently demonstrate that many pond farms—especially smaller ones—cannot sustain themselves economically in the long run. Consequently, maintaining effective pond management and preserving biodiversity requires continuous financial support from society (AG Lasner et al. 2020; NASTAQ 2020), underscoring the need for robust biodiversity governance, which is discussed in the next section.

## Governance for biodiversity-rich pondscapes

### Multiple actors, multiple instruments

Biodiversity governance refers to the institutions, structures, and processes that determine how and by whom decisions affecting biodiversity are made (Bennett and Satterfield 2018). With governments traditionally being the most important decision-makers in conservation, alternative ways of governing involving new actors and mechanisms are becoming increasingly relevant. This also impacts the use of policy instruments, which are structured activities aimed at achieving long-term environmental goals. Policy instruments can be categorised into four categories: legal and regulatory instruments, rights-based instruments and customary norms, economic and financial instruments, and social and cultural instruments (Ring et al. 2018). In this article, we focus on two of these categories, legal and regulatory instruments as well as economic and financial instruments.

The conservation of aquatic biodiversity and wetlands has long been characterised using legal and regulatory instruments, such as the US Clean Water Act or the EU Water Framework Directive. The failure of these efforts to halt and reverse biodiversity loss has led to a growing interest in economic instruments, such as payments for environmental services, biodiversity offsets, or biodiversity credits (Ring and Schröter-Schlaack 2011; Schwerdtner Máñez and Clifton 2025).

Payments for environmental services (PES) have played a major role in global conservation policy over more than two decades (Porras et al. 2013; Wunder 2015; Clifton and Schwerdtner Máñez 2025). They offer monetary incentives to individuals or communities to voluntarily adopt behaviours that are not legally obliged, and which target and improve the provision of well-defined ecosystem services that they would otherwise have been economically unviable to provide (Sommerville et al. 2009; Muradian et al. 2013). PES are currently the most significant instrument for financing the maintenance of pond landscapes, as they provide payments for environmental services to pond farmers and other actors preserving the pond landscapes. In several EU countries, such as Germany, Austria and Poland, PES for pond farming are implemented. Especially when co-financed with EU-fisheries funds, they mostly only compensate for the additional costs of pond farmers of the relevant management activities, but do not reward the actual benefits in terms of the wider services of these measures provided to society. However, many of today’s cultural landscapes shaped by extensive agricultural or aquaculture production on marginal lands, such as high nature value farm- and grasslands (Keienburg and Prüter 2006; Matzdorf et al. 2010), or extensively managed pondscapes (Müller-Belecke 2023b), are costly to maintain and often not financially viable to manage. Here, farmers depend on receiving payments for the environmental services they provide to society, and this means assessing the wider benefits for biodiversity conservation and ecosystem services provision as well as considering design rules for successful governmental PES (Meyer et al. 2015; Albert et al. 2017).

As part of its offset policy, the USA pioneered the concept of commercial wetland mitigation banking in 1991, creating a market for privately owned “wetland ecosystem services” such as duck habitat, flood protection and biodiversity (Robertson 2004). This has led to the development of conservation banking: Landowners who manage their land for conservation purposes can establish a conservation bank and are granted credits that can be purchased by developers (White et al. 2021). Freshwater ecosystems are included in several offsetting frameworks (Hermoso and Filipa Filipe 2021), and in England, standardised pond units can be purchased to offset development under the Biodiversity Net Gain approach (DEFRA 2023).

Biodiversity credits are the latest addition to the portfolio of economic instruments in conservation policies. While they focus on the conservation and enhancement of biodiversity, biodiversity credits are not linked to specific operations. As a result, they have the potential to contribute to the long-term conservation of natural habitats and the restoration of degraded ecosystems (Schwerdtner Máñez and Clifton 2025). Freshwater ecosystems are included in only a small number of the 53 biodiversity credit schemes currently in operation or under development (see Bloomlabs 2025), for example, as aquatic ecosystem function credits (Willamette Partnership 2025).

Recent research on the sustainable financing of ponds has identified a range of potential instruments for consideration, including user fees, philanthropic contributions, crowdfunding, environmental subsidies, green bonds and loans, or grants (McDonald et al. 2023). As awareness of the importance of ponds and pondscapes grows, these instruments may gain increased relevance.

### Conserving the Lusatian pondscape: Environmental policy instruments at play

Pond aquaculture in Lusatia is shaped by a mix of policies, with a legal framework rooted in European legislation, further specified by national and state laws (Schwerdtner Máñez and Ring 2021). Key areas include conservation, fisheries, water, state aid, and regulations on animal welfare and disease control. The most relevant policy instruments for maintaining pondscapes as biodiversity-rich cultural landscapes focus on providing financial support for biodiversity conservation and the provision of environmental services. They also include measures to deter and prevent damage by protected species, along with compensation for any resulting harm (see Table 1).

**Table 1:** Policy instruments for conserving pondscapes in Brandenburg and Saxony (economic instruments emphasised in italics)

	Brandenburg	Saxony
Compensation for nature conservation measures	*Funding guideline for aquaculture and inland fisheries, Sect. 2.2.3 compensation for environmental services in carp pond aquaculture*	*Funding guideline for pond management and nature conservation*
Regulated deterrence and damage prevention for protected species	Cormorant ordinance	Cormorant ordinance
	Beaver ordinance	Hunting ordinance (with a culling quota for grey herons)
	*Funding guideline for prevention measures to protect against beaver damage*	
Damage compensation	*Compensation guideline for damage caused by protected species in pond farms*	*Hardship compensation regulation*

The EU Common Fisheries Policy (CFP) oversees the market and financial aspects of aquaculture, with funding through the European Maritime and Fisheries Fund (EMFF), which supported nature conservation efforts in pond farming from 2014 to 2023. This was replaced by the European Maritime, Fisheries and Aquaculture Fund (EMFAF), providing 6.1 billion € to co-fund national projects from 2021 to 2027 (Council of the EU 2020). To access funding, member states must have a National Aquaculture Strategic Plan.

Germany’s operational programme prioritises the preservation of pond aquaculture and pondscapes in its fisheries policy (Germany—Operational Programme for EMFAF 2021–2027), supporting state-level initiatives. Payments for environmental services in aquaculture mirror agri-environmental schemes under the Common Agricultural Policy. Fish farmers receive compensation for income losses or additional efforts to implement environmental measures, typically for five years. In Lusatia, these measures vary between Brandenburg and Saxony.

In Brandenburg, the new EMFAF-based *Funding Guideline for Aquaculture and Inland Fisheries* provides compensation for environmental services in carp pond aquaculture. Maintenance Plan A offers a basic premium of 250€/ha/a for extensive fish production and pond maintenance measures. Maintenance Plan B, co-funded by federal funds, requiring consultation with nature conservation authorities, includes additional measures with funding obligations that can be combined, e.g. related to damming and stocking limitations. The compensation amount varies between measures in a range from 29 to 367€/ha/a (MLUK 2024). With the new guideline being in place since May 2024, funding amounts were significantly increased compared to the previous period, where similar measures were reimbursed only at an overall maximum of 150€/ha/a.

Since October 2022, Saxony has implemented the EMFAF-based *Funding Guideline for Pond Management and Nature Conservation.* The funding programme includes two parts: Part A, co-funded by EMFAF and Saxony, offers support for conservation-friendly pond management and measures prescribing, e.g. yield limits and/or excluding the stocking of predatory fish species. Part B, co-funded by federal funds, covers extensive fish production measures of non-aquaculture actors and maintenance measures for ponds without fish stocking. Organic carp production can receive additional financial support. The amount of compensation payment ranges from 205 to 820€/ha/a (SMEKUL 2023).

Furthermore, Brandenburg and Saxony have implemented regulations to manage conflicts with protected species (Table 1). Brandenburg’s *Cormorant Ordinance*, in place since 1999, allows deterrent shooting outside nature reserves to prevent significant fishery damage and breeding colony establishment. Grey herons and great egrets require special permits for control, while a beaver ordinance permits dam and lodge removal in commercial fishponds and, in some cases, culling. The aquaculture and fisheries guideline subsidises protective measures like nets and fences, with separate funding available for beaver damage prevention.

Saxony’s *Cormorant Ordinance* permits culling and breeding colony prevention to protect the fisheries sector and native wildlife. The *Saxon Hunting Ordinance* allows grey herons to be hunted within 200 m of ponds to prevent fishery damage, though this is subject to a quota.

In addition to these regulatory measures, damage compensation plays a key role in balancing nature conservation with the interests of pond farmers (Rabe von Pappenheim 2023). These payments reflect the principle that protecting species like cormorants and otters is a societal objective, while the costs are largely borne by private individuals. Strict protection measures also limit options for damage prevention. Compensation can help mitigate conflicts and foster acceptance of protected species (Schwerdtner and Gruber 2007).

The EU has no uniform regulations on compensating damage from protected species, leaving it to individual member states. Germany’s *Framework Compensation Guideline for Damage Caused by Protected Animals in Fisheries and Aquaculture* enables states to create their own compensation rules, independent of state aid law (RRL 2023).

In Brandenburg, the *Compensation Guideline for Damage Caused by Protected Species in Pond Farms* covers damage to commercial carp stocks and beaver-related damage, particularly to inlet and outlet structures, embankments, and dams. Compensation for damage to carp stocks is calculated based on a flat rate for each age group and production area. Beaver damage compensation is determined by an official beaver consultant, with affected aquaculture businesses eligible for up to 100% compensation for direct damage.

In Saxony, the *Hardship Compensation Regulation* provides financial relief for damages caused by wild animals. It covers up to 60% of total losses, with the possibility of increasing compensation to 80% under special site conditions.

### The TeichLausitz project: Assessing governance and charting a path to solutions

The “TeichLausitz project” (2021–2025) develops recommendations for supporting pond conservation and its biodiversity. An evaluation of the regulations and compensation schemes in Brandenburg and Saxony related to damage caused by protected species found existing policies inadequate to sufficiently reduce farm damage or resolve conflicts with fish farmers. Brandenburg offers better support for dealing with beaver damage, including easier removal and compensation for non-fish-eating species, while Saxony allows more freedom to manage cormorants and offers hunting options for grey herons (Rabe von Pappenheim 2023).

With the new EMFAF funding period, Saxony revised eligible areas for certain measures to align with Natura 2000 monitoring and evaluation results, sparking conflicts with fish farmers. They criticised their exclusion from the process and the lack of scientific basis for certain measures, such as restricting maximum annual yield to 400 kg/ha despite limited evidence that this positively effects habitat or priority species preservation. Farmers also opposed treating each pond as separate, ignoring their pond group-based management approach and specific year-class management schemes.

To address concerns over recent funding guideline changes, the TeichLausitz project held a workshop in March 2023 with 54 participants, including pond farmers, fishery and environment protection authorities, and conservation groups. While Saxony’s new guideline was already published, Brandenburg’s was not, making Saxony the primary focus. In the first part of the workshop, a shared information base was established, with representatives from environmental ministries and conservation authorities outlining the process of developing the new funding programme, while pond farmers discussed its impact on pond farming. The findings highlighted a disconnect: policymakers, driven by EU legislation, prioritised habitat conservation, particularly in Saxony, while fish farmers felt excluded, fearing their businesses were at risk, although key to maintaining biodiversity and habitat preservation.

**Fig. 2:**
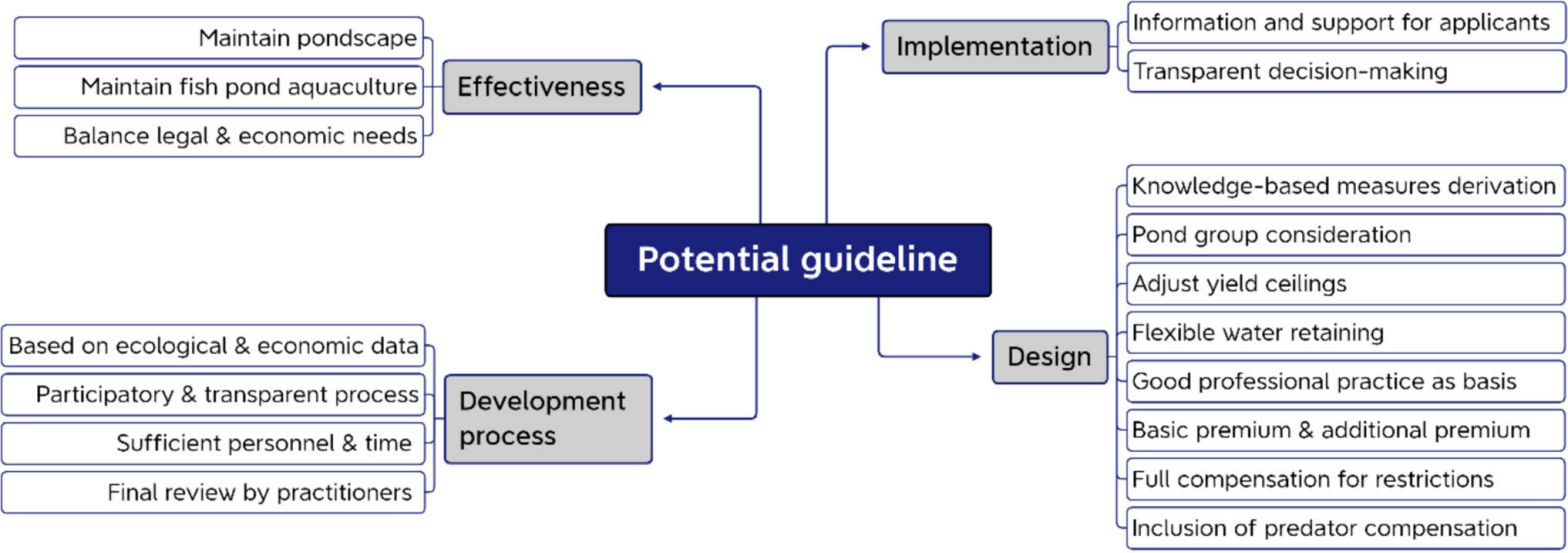
Potential guideline for supporting the maintenance of pondscapes

In the second part of the workshop, participants developed a framework for a future guideline to conserve the pond landscape and ensure the economic sustainability of fish farms. Using the Min Specs method (Lipmanowicz and McCandless 2013), they identified the essential “must dos” and “must not dos” for success. First, they compiled a list of all necessary rules. Then, four breakout groups (10–12 participants each) tested each rule against the purpose statement, discarding non-essential ones. Finally, the groups compared their findings in a plenary session and agreed on a final list, organised into four key categories: (1) effectiveness, (2) development process, (3) implementation, and (4) design (Fig. 2).

1. “Effectiveness” refers to a guideline’s ability to preserve pond landscapes and biodiversity while sustaining pond aquaculture. This requires balancing legal conservation requirements with the economic needs of fish farmers.
2. The “guideline’s development process” must be grounded in robust ecological and economic data to ensure informed funding decisions. A participatory and transparent process is essential for acceptance and integrating diverse knowledge effectively. Participants stressed the need for adequate personnel and time, while pond farmers highlighted final approval from practitioners as key to the guideline’s success.
3. “Implementation” refers to the practical application of the guideline. Participants emphasised the need for clear information, support in drafting applications, and transparency in decision-making regarding approved measures and accepted applications.
4. To “design” the guideline, several key factors were agreed upon. Measures should be grounded in solid knowledge, and a comprehensive assessment of pond groups is essential, considering factors like pond positioning, water supply, and multi-year production cycles. Adjusting yield ceilings and implementing flexible water retention strategies were seen as crucial for economic viability. The principle of “good professional practice” (Füllner et al. 2007) should serve as a foundation.

Current financial support mainly covers income losses and additional costs but does not sufficiently reward the environmental services provided by fish farmers to society. As a result, funding lacks the necessary incentive for long-term engagement in fish farming. Participants agreed on the need for a sufficient basic premium, with supplementary payments for additional efforts. Any imposed restrictions should be fully compensated, and predator compensation should be integrated into the funding programme rather than managed separately.

## Ways forward for conserving European pondscapes

Pondscapes provide an excellent case study of a cultural landscape in which biotopes for biodiversity are co-produced through the interaction between human management and natural processes over time. The conservation of these habitats presents a significant opportunity to mitigate threats to freshwater ecosystems and hold the decline of freshwater biodiversity on a global scale (Hill et al. 2017). The new paradigm for conservation emphasises the necessity for continuous and intensive care to conserve man-made habitats and their biodiversity (Kueffer and Kaiser-Bunbury 2014). This situates humans in the role of “guardians of biodiversity” (Teneggi and Zandonai 2017). It is crucial to align conservation objectives with the economic needs of those responsible for managing these landscapes. Policies that treat biodiversity as an isolated value, detached from the economic realities of land managers, are unlikely to succeed (Riechers et al. 2021; Bobiec et al. 2025).

The regional focus of our article comes with certain limitations, meaning that not all findings can be directly transferred to other European pond landscapes. Nevertheless, it is evident that traditional carp pond farming across Europe faces similar challenges: restrictive legal requirements, rising costs, and increasing production losses due to predators equally burden pond operations in many regions.

The protective status of many ponds limits the options available to pond farmers when selecting and applying production schemes, particularly in terms of economic feasibility. Financial support for farmers managing ponds to maintain habitats of priority importance and their biodiversity should therefore be viewed as a societal obligation and a fair and necessary remuneration. While current instruments only compensate for the additional effort required for certain measures or the income lost due to restrictions, this would require a genuine reward for the environmental services provided by pond farmers. Such a “pond premium” would need to be high enough to make pond farming profitable in the long term, thereby creating a real incentive for its continuation. Future research should provide insights on the amount of the pond premium, for example, based on economic evaluation studies of environmental services provided by pond farming.

Integrating the needs and expertise of pond farmers into policy instrument design is essential for ensuring both effectiveness and broad acceptance. Co-designing measures with stakeholders not only builds support, it also enhances ecological and economic efficiency (Hölting et al. 2022). The TeichLausitz project demonstrates that such collaborative approaches can reconcile diverse stakeholder demands and create a unified framework for policy instruments. To further support this, future social research is needed to examine the perceptions and motivations of pond farmers in relation to their increasing dependence on subsidies.

Pondscapes are receiving growing attention for their ecological and cultural importance, which might prompt an expansion of policy instruments—including innovative tools such as biodiversity credits or crowdfunding. However, only a policy mix that balances economic needs with conservation goals will ensure these unique landscapes and their biodiversity endure for future generations.
